# Estimation of bone mineral density with computed tomography performed in radiotherapy planning

**DOI:** 10.1016/j.phro.2026.100986

**Published:** 2026-05-03

**Authors:** Julia Götstedt, Frida Ljung, Jenny Nilsson, Erik Pettersson, Sofia Heyman

**Affiliations:** a Department of Medical Radiation Sciences, Institute of Clinical Sciences, Sahlgrenska Academy, University of Gothenburg, Gothenburg, Sweden; b Department of Therapeutic Radiation Physics, Medical Physics and Biomedical Engineering, Region Västra Götaland, Sahlgrenska University Hospital, Gothenburg, Sweden; c Department of Oncology, Institute of Clinical Sciences, Sahlgrenska Academy, University of Gothenburg, Gothenburg, Sweden; d Department of Oncology, Region Västra Götaland, Sahlgrenska University Hospital, Gothenburg, Sweden

**Keywords:** Osteoporosis, Trabecular bone, dual energy X ray absorptiometry, Phantom, Internal calibration, Breast cancer

## Abstract

**Background and purpose:**

Cancer patients are at increased risk of impaired bone health. The aim of this study was to develop a method for estimating bone mineral density (BMD) from computed tomography (CT) images acquired for radiotherapy (RT) planning.

**Materials and methods:**

We calculated BMD (mg/cm^3^) in trabecular bone specimens (n = 28) with known elemental composition and simulated their radiodensity at 71 keV, corresponding to 120 kV commonly used in CT scanning. A linear translation function from CT number to BMD was derived and validated with phantom measurements.

A set of 178 patient scans acquired with two different CT scanners in RT planning was retrieved. Trabecular bone was defined by placing a spherical volume of interest (VOI) centrally in the 9th-12th thoracic vertebrae. Interobserver variability was evaluated for VOIs delineated by three independent observers. Estimation of BMD for patient data using the translation function was validated against BMD estimated by phantom and internal calibration. Scanner differences were assessed by phantom measurements.

**Results:**

The derived translation function (BMD = 0.7161 × CT number − 1.212; R = 0.99) agreed in the patient data set with the spine phantom calibration (mean difference 8.7 mg/cm^3^ with confidence interval (CI) 1.4−16, R = 1.0) and internal calibration (mean difference -1.5 mg/cm^3^, CI -13.1−10.2, R = 0.99). The delineation method showed a high interobserver agreement (intraclass correlation coefficient 0.99, CI 0.99−1).

**Conclusions:**

A method for reliable BMD assessment from RT CT images was successfully developed and validated. Future studies should establish clinically relevant BMD cut-off values.

## Highlights

• Bone mineral density on computed tomography (CT) in radiotherapy planning.
• Theoretically derived function: Bone mineral density = 0.7161 × CT number − 1.212.
• The method is robust to inter-scanner and inter-observer variability.
• Function validated using both phantoms and internal calibration for 152 patients.

## Graphical abstract

**Figure:**
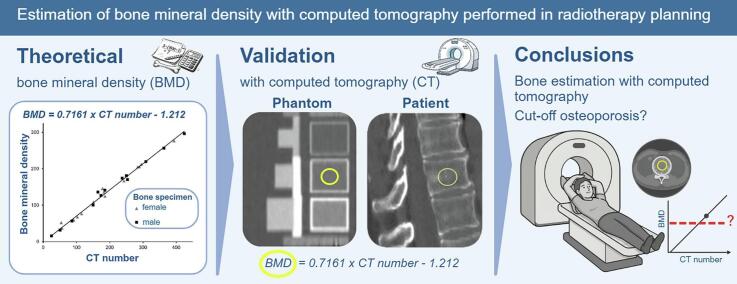

## 1 Introduction

Cancer patients are at risk of poor bone health for multiple reasons, e.g. immobilization, malnourishment and prolonged steroid therapy. In addition, some oncological treatments, such as endocrine therapy used in breast and prostate cancer may cause osteoporosis [1], [2]. Moreover, underlying osteoporosis can increase the risk of therapy-related side effects such as radiotherapy-induced insufficiency fractures [3], [4], [5]. Finally, it has been suggested that low bone mineral density (BMD) may enhance tumour seeding in bone or accelerate progression of bone metastasis [6], [7], [8].

Dual energy X-ray absorptiometry (DXA) is the established standard to estimate areal BMD (g/cm^2^) to assess risk of osteoporosis, however, it has several limitations [9]. For one, it is a two-dimensional (2D) assessment, limiting the possible sites of examination as it will be sensitive to overlapping structures. It is also sensitive to patient body size, imaging artefacts and patient motion. Furthermore, the result of a DXA examination depends on DXA machine vendor, calibration, and the reference data used. As an alternative to DXA, the measurement of BMD has been performed using computed tomography (CT), which has the advantage that a bone structure can be assessed regardless of anatomical location [10], [11], [12], [13], [14], [15]. However, radiation exposure is significantly higher with CT compared to DXA, making it less suitable for screening of healthy subjects. Moreover, there is no established method to translate CT numbers (Hounsfield units; HU) into volume BMD (mg/cm^3^) and we lack reference intervals for normal BMD measured by CT.

Approximately 50% of all cancer patients will receive radiotherapy (RT) at which a CT scan is performed for treatment planning and dose calculations [16]. High demands are put on the accuracy of these CT images regarding shape and density which makes CT in RT especially suitable for opportunistic assessments of BMD.

The overall aim of the study was to develop a universal method for BMD estimation in trabecular bone depicted with CT in RT planning. In this study, we aimed to derive a method to translate CT number to BMD on theoretical grounds, where radiodensity and BMD were based on tabulated data for elemental composition, mass attenuation, and density for reference bone specimens. The derived function was validated with phantom scans and in a study population.

## 2 Materials and methods

### 2.1 Development of a CT based method for BMD estimation

A method to translate HU to BMD was developed. Male and female bone specimens (n = 28) from 14 different locations in the skeleton were selected to cover a range of different bone densities (Supplementary material A A and Table S1-2). Radiodensity and BMD of the reference bone specimens were plotted, and linear regression was used to obtain a translation function for radiodensity to BMD (mg/cm^3^).

The CT scanners used were either SOMATOM go.Open Pro (Siemens Healthineers, Germany) or Aquilion LB (Toshiba Medical Systems Corporation, Japan). The CT scans were performed according to standard protocols (Supplementary material B).

**Fig. 1:**
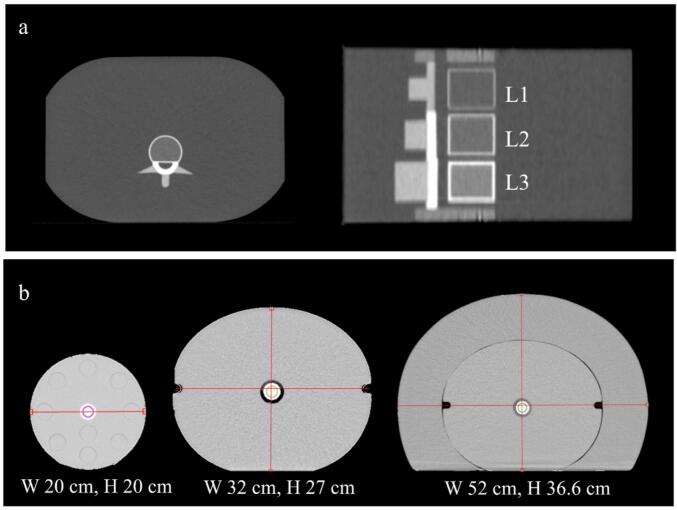
Phantom validations. a) A spine phantom was used for validation of the translation function. The shape of the vertebral inserts (L1, L2, L3) are shown on a transverse view and sagittal view, respectively. The inserts had specified BMD, ranging from 50 mg/cm^3^ (L1) to 200 mg/cm^3^ (L3). The spine phantom was scanned with both CT (SOMATOM) and DXA. b) Phantoms with three different sizes (small, medium, large) were used and scanned with CT (SOMATOM). W, width; H, height; BMD, bone mineral density; CT, computed tomography; DXA, dual-energy absorptiometry.

A spine phantom (European Spine Phantom; ESP) by QRM GmbH (Germany) was used to validate the derived translation function from HU to BMD [18], [19]. The ESP had space for three inserts with different densities replicating the shape of the first, second and third lumbar vertebrae (L1, L2, L3), Fig. 1. The phantom was scanned with the SOMATOM CT using a standard protocol for a thoracic scan. On the CT scans, a spherical volume of interest (VOI) was placed in the centre of each insert in which the radiodensity was translated to BMD using the function derived in this study. Also, a calibration curve was derived from the ESP data as the linear relationship between the measured radiodensity and the manufacturer–specified BMD, and this curve was then used to compare the BMD of the study population estimated using the derived translation function.

Further, the same ESP was scanned using a DXA lumbar spine examination protocol (Horizon A S/N 200786, Hologic, Inc., USA) to compare with the performance of the derived CT-based BMD estimation method.

The CT number variation due to body size was evaluated using three configurations of two electron density phantoms (Gammex 1467 & CIRS 062A; Sun Nuclear, USA) (Fig. 1): small (W 20 cm, H 20 cm), medium (W 32 cm, H 27 cm), and large (W 52 cm, H 36.6 cm). Four inserts of bone-equivalent material, containing calcium carbonate (CaCO_3_), were used to represent the range from trabecular to cortical bone (Sun Nuclear). The manufacturer-designated names of these inserts were HE Inner Bone, CB2 + 30% CaCO_3_, CB2 + 50% CaCO_3_, and HE Cortical Bone. The phantoms were scanned using four routine CT protocols for pelvis, thorax, head and neck, cranium and spine and the scans were repeated three times for each scan protocol for each insert centrally placed within the phantoms. For each CT scan of the different phantom sizes, CT numbers were derived as the mean value in a cylindrical VOI placed in the central part of the phantom inserts and translated to BMD using the derived function.

Scans obtained from constancy tests on Aquilion and SOMATOM using an electron density phantom (CIRS 062A; W 33 cm, H 27 cm) and inserts replicating bone with different densities were retrieved. The CT protocols used were optimized for thoracic scans. For each of the CT scans from different CT scanners, the CT numbers of the phantom inserts were translated to BMD and compared.

### 2.2 Method validation on study population

**Fig. 2:**
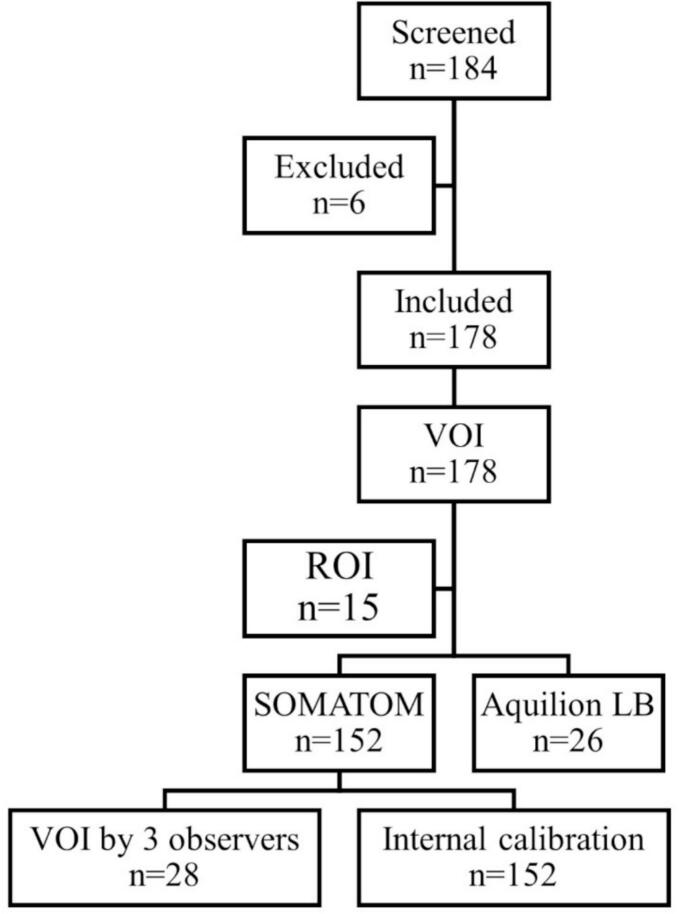
Flowchart of the study population. Patients receiving RT after breast cancer surgery were screened for inclusion in the study (n = 184). Patients receiving RT with palliative intent were excluded (n = 6). Delineations were made on CT scans from 178 patients: VOI of vertebral body/trabecular bone of Th4-L1, fat, blood, air. In addition, ROIs in Th4-L1 were defined on CT scans from 15 of the 178 patients. CT scans from SOMATOM (n = 152) and Aquilion LB (n = 26), respectively. RT, radiotherapy; CT, computed tomography; VOI, volume of interest; Th4; 4 h thoracic vertebra; L1; first lumbar vertebra; ROI, region of interest.

We included 178 female patients (median age 68 y) that had undergone CT for planning of adjuvant radiotherapy for breast cancer from 1st March 2020 – 1st May 2025 (SOMATOM n = 152; Aquilion n = 26) (Fig. 2). We selected this patient group because breast cancer patients are treated at all RT units, they are CT scanned without contrast, and the scan includes a large part of the spine in a highly standardized manner. The study was conducted with respect to the General Data Protection Regulations (GDPR) and was ethically approved by the Swedish Ethical Review Authority (registration number 2024–03650-01).

**Fig. 3:**
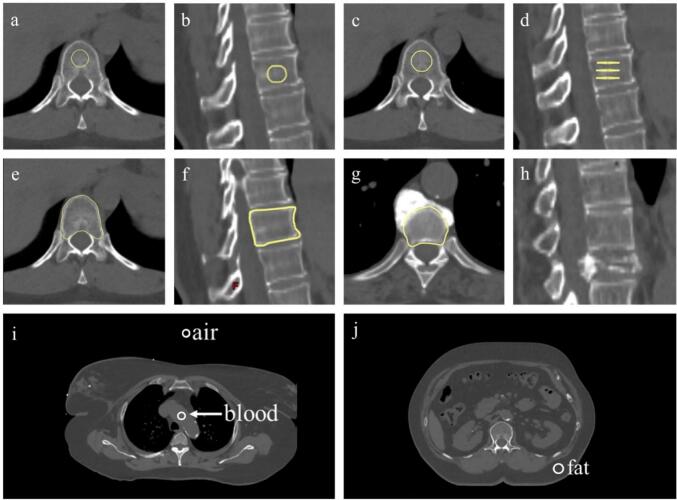
Delineation of bone, air, blood and fat. a-b) A spherical VOI is placed in the centre of Th12; c-d) three planar ROIs are placed within Th12; e-f) the vertebral body of Th12 is delineated including both trabecular and cortical bone; g) the vertebral body is delineated excluding osteophytes; h) the vertebra (Th9) is compressed and excluded. i-j) For purpose of internal calibration, a VOI is placed in air, in the blood pool within the aortic arch and in fat tissue at the lower back. VOI, volume of interest; Th12, 12th thoracic vertebra; ROI, region of interest; Th9, 9th thoracic vertebra.

The patients were scanned in the supine position with both arms above their heads. All vertebrae (C2-L1) included in the patient CT scan were evaluated. Due to the curvature of the spine and size of the vertebrae, the cervical and upper thoracic spine was excluded from further analysis. Three methods for obtaining CT numbers from the vertebrae were tested (Fig. 3). In method 1, a circular area (region of interest; ROI) was delineated at three separate transverse planes in each vertebra (D 0.5–1.5 cm) Th4-L1 in 15 CT scans. In method 2, a spherical VOI was placed in the centre of each vertebra (D 0.5–1.5 cm) Th4-L1 on 15 scans and in Th9-Th12 on a further 163 scans. Three observers made independent delineations for Th9-Th12 on 28 of the CT scans. In method 3, the vertebral body of Th9-Th12 (trabecular and cortical bone) was delineated on 178 scans. Areas of focal osteosclerosis or osteolysis were avoided. Vertebrae with distinct degenerative changes, such as vertebral compression fractures, were excluded from analysis. The CT number of each vertebra was the mean of the three ROIs or the mean in each VOI. The radiodensity of the ROI/VOIs was translated into BMD using the translation function.

To validate the translation function, theoretically determined BMD was compared with BMD determined using internal calibration for 152 patients scanned with the SOMATOM CT [20]. Air, fat and blood were chosen as calibration materials, and their mass densities and attenuation coefficients were retrieved from National Institute of Standards and Technology (NIST) [21]. The mass density and elemental composition of pure bone mineral (calcium hydroxyapatite, CaHA) are known [22]. A spherical VOI (D 0.5–2 cm) was placed in external air, in the aortic blood pool and in fat tissue either in the lower back or clavicular fossae (Fig. 3). Trabecular bone was defined by placing a spherical VOI in the centre of the vertebra as described above. The BMD in Th9-12 was calculated by summing the mass attenuation coefficients for each of the individual components weighted according to their proportion in bone mineral (Eq. (1):

(1) $$\mathrm{BMD}=\rho_{\mathrm{CaHA}}=\frac{{\left(\frac{\mu }{\rho }\right)}_{1}\rho_{1}\frac{CT_{B}-CT_{2}}{CT_{1}-CT_{2}}+{\left(\frac{\mu }{\rho }\right)}_{2}\rho_{2}\frac{CT_{B}-CT_{1}}{CT_{2}-CT_{1}}-{\left(\frac{\mu }{\rho }\right)}_{w}\rho_{w}}{{\left(\frac{\mu }{\rho }\right)}_{\mathrm{CaHA}}-{\left(\frac{\mu }{\rho }\right)}_{w}\frac{\rho_{w}}{\rho_{\mathrm{CaHA}}}}$$

where *ρ_CaHA_* = mass density of CaHA; *µ/ρ* = mass attenuation coefficient; *ρ* = density; CT_B_ = CT number of the bone structure studied (mean of CT number in VOI Th9/10/11/12); CT_1_ = CT number of internal calibration material 1 (fat or blood); CT_2_ = CT number of internal calibration material 2 (air); *w* = water.

### 2.3 Statistical analysis

Quantitative data were presented as median, mean or mean difference values with 95% confidence interval (CI), standard deviation (SD), standard error of mean (SEM), range or Bland-Altman limits of agreement (±1.96 SD). Bland-Altman analyses were performed to visualize correlation between data and to evaluate method agreement. Linear regression and the Pearson’s correlation coefficient (R) were used to examine the relationship between variables. Inter-observer reliability was evaluated by determining the intraclass correlation coefficient (ICC) where ICC > 0.9 was considered an excellent agreement. P-values < 0.05 were considered statistically significant.

## 3 Results

**Fig. 4:**
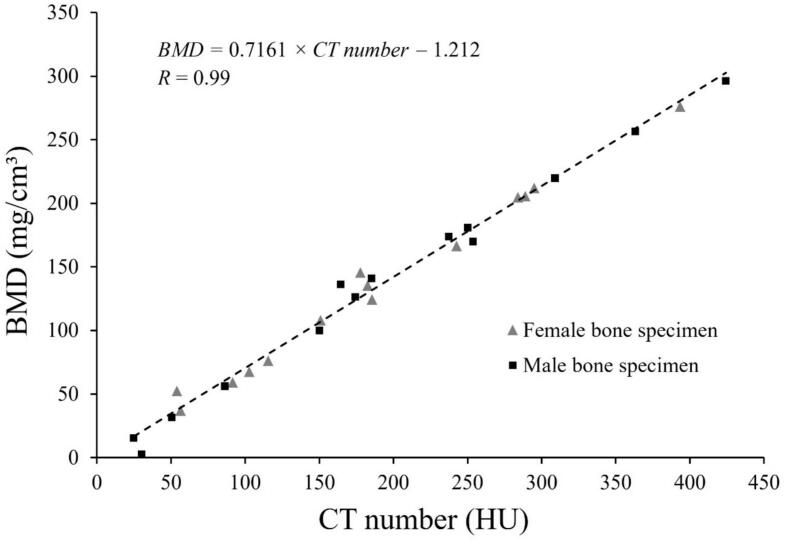
A linear regression showing BMD as a function of CT number. The analysis was based on trabecular bone specimens (n = 28) from 14 different localisations in the skeleton from both men (squares) and women (triangles). BMD and CT numbers were theoretically derived based on elemental composition.

Radiodensity and BMD were theoretically determined for the trabecular bone specimens (n = 28). The BMD (mg/cm^3^) was plotted as a linear function of CT number (Fig. 4), Eq. (2):

(2) BMD=0.7161×CTnumber-1.212,R=0.99

The translation function (Eq. (2) was used to estimate BMD of the inner core of the vertebral phantom inserts (Table 1). A strong agreement was found between the manufacturer’s specification and BMD estimated using the translation function with a mean absolute difference of 2.9 mg/cm^3^ (SEM 2.3), R = 1. Best agreement was found for the BMD range 50–100 mg/cm^3^. A weaker agreement was shown between measured areal BMD using DXA.

**Table 1:** Phantom validations. a) BMD estimated in vertebral bone inserts (L1, L2, L3) in a spine phantom using SOMATOM CT and DXA, compared with the manufacturer’s specification within brackets. Data are presented as mean BMD (±SD) from repeated scans (n = 3). b) Mean BMD ± SD for bone inserts of varying densities within phantoms with different dimensions from CT scans run with four different protocols. BMD, bone mineral density; L1/L2/L3, lumbar vertebra 1, 2, 3; CT, computed tomography; DXA, dual energy X-ray absorptiometry; SD, standard deviation.

**a) BMD estimated using CT or DXA**			
**Spine phantom** (ESP)	**SOMATOM CT** BMD (mg/cm^3^)	**DXA** areal BMD (mg/m^2^)	
L1	49.9 ± 0.8 [50]^a^	510 ± 2 [500]	
L2	98.8 ± 0.3 [100]	990 ± 0.1 [1000]	
L3	192.5 ± 1 [200]	1430 ± 2 [1500]	

**b) BMD estimated using phantoms with different dimensions (cm)**			

**Electron density phantoms** (Sun Nuclear)	**SOMATOM CT, BMD (mg/cm^3^)**		
**Solid water phantoms (cm)**	**Small** (W 20, H 20)	**Medium** (W 32, H 27)	**Large** (W 52, H 36.6)

HE Inner Bone	227 ± 2	228 ± 2	254 ± 5
CB2 + 30% CaCO_3_^b^	353 ± 2	358 ± 2	396 ± 5
CB2 + 50% CaCO_3_^c^	658 ± 3	659 ± 3	714 ± 9
HE Cortical Bone	1117 ± 4	1114 ± 4	1199 ± 14

^a^the manufacturer’s specification is shown within brackets; ^b-c^CaCO_3_ − 30% alt 50%, concentration of calcium carbonate 30% alt 50%. W, width; H, height [cm].

An overall high agreement in estimated BMD was observed for bone inserts in different sized phantoms irrespective of the CT protocol chosen (Table 1). For the small and medium sized phantoms, a deviation in BMD up to 4 mg/cm^3^ (SD) was seen for all bone inserts and when scanned with different CT protocols. For the bone insert corresponding to trabecular bone (HE Inner Bone) the deviation was only 2 mg/cm^3^ (SD) for both small and medium sized phantoms. For the largest phantom slightly larger deviations (SD 5–14 mg/cm^3^) were seen for the inserts when scanned with different CT protocols, especially when scanning bone inserts with higher density (CaCO_3_ 50%, HE Cortical Bone).

A high agreement in mean CT numbers and translated BMD was seen when scanning the same phantom with bone inserts using two different CT scanners, SOMATOM and Aquilion, with CT protocols optimized for thoracic scans. The inter-scanner variability for the four bone inserts, expressed as the mean absolute difference, was 5.9 mg/cm^3^ (SEM 2.4). The BMD was overall similar for the different CT scanners, as shown by the linear relationship (Eq. (3) with a coefficient close to one (1.009) and a low constant difference (−4.32).

(3) $$\mathrm{BM}D_{\mathrm{SOMATOM}}=1.009\times BMD_{\mathrm{Aquilion}}-4.32,R=1.0$$

However, the intra-scan variability was higher in the CT scans performed with Aquilion. This was reflected by larger variations in CT numbers (HU) within the VOI of the inserts when scanned by Aquilion (mean variation 76 HU) compared to SOMATOM (mean variation 16 HU), especially at higher densities.

The mean CT number for each vertebra was between 6–16 HU higher when using a spherical VOI compared to ROIs. The Pearson correlation coefficient was 0.98 (p < 0.001), indicating a strong correlation between the two methods.

It was observed that BMD decreases in the craniospinal direction of the spine and that anatomical variations of single vertebrae can influence the results. Therefore, further analyses were based on radiodensity in the trabecular bone in Th9-Th12 by placing a sphere (D 0.5–1.5 cm) in the centre of each vertebra.

The interobserver variability was assessed and found to be excellent with an ICC for single measures of 0.98 (CI 0.97–0.99) and for average measures 0.99 (CI 0.99–1).

Radiodensity was measured in trabecular bone of Th9-Th12 (VOIs) on 178 CT scans (SOMATOM = 152; Aquilion = 26). The CT numbers were translated into BMD using the translation function (Eq. (2) and the spine phantom calibration function, and the results correlated well (R = 1.0), even though BMD estimated with the calibration curve tended to be higher with a mean difference 8.7 mg/cm^3^ (CI 1.4–16). Using Eq. (3), Aquilion measurements were adjusted to match SOMATOM, yielding a mean difference of 3.4 mg/cm^3^ (CI 3.0–3.9) between the estimated and adjusted BMD. In this real-world population of women receiving adjuvant RT for breast cancer the median BMD (Th9-Th12) was 101 mg/cm^3^ (range 33–206) according to our method.

**Fig. 5:**
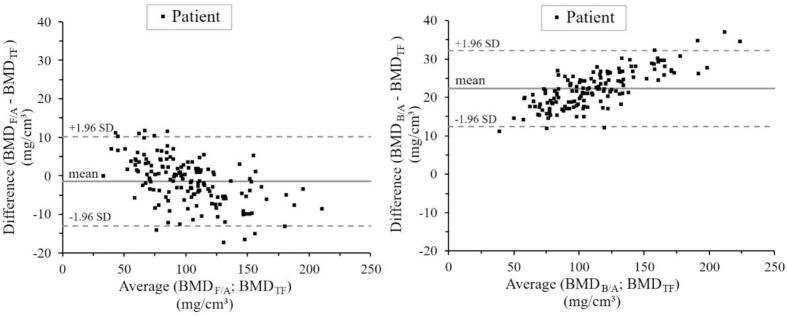
Comparison of BMD calculated with translation function or internal calibration. A VOI was placed in Th9-Th12 on CT scans (SOMATOM) from 152 patients (black squares). BMD was estimated using the translation function (BMD_TF_) and internal calibration with fat/air (BMD_F/A_) or blood/air (BMD_B/A_) as materials. The solid grey line indicates mean difference, the dashed grey lines are the mean ± 1.96 SD. BMD_TF_ and BMD_F/A_ showed high agreement with a mean difference of -1.5 mg/cm^3^ (CI -13.1−10.2), whereas blood/air systematically rendered higher BMD with a mean difference of 22.3 mg/cm^3^ (CI 12.4–32.2). BMD, bone mineral density; TF, translation function; F/A, fat/air; B/A, blood/air; SD, standard deviation; CI, confidence interval.

Radiodensity of Th9-Th12 (spherical VOI) was measured and translated to BMD using the internal calibration method with fat/air or blood/air as calibration materials. Calculation of BMD using blood/air yielded a higher BMD than using the combination of fat/air as calibration materials (Fig. 5).

The BMD estimated with the derived translation function correlated with BMD estimated with internal calibration using fat/air (R = 0.99, p < 0.001) with a mean difference of -1.5 mg/cm^3^ (CI -13.1−10.2).

## 4 Discussion

The present study describes a reproducible method for estimating BMD in the lower thoracic spine from RT planning CT. The CT numbers showed a strong linear relationship with BMD, validated for both phantom measurements and internally calibrated estimates. The method demonstrated low interobserver variability and remained robust across scanner types, protocols, and patient sizes. The translation function was theoretically derived using high-quality data from International Commission on Radiological Protection (ICRP) reports and the National Institute of Standards and Technology (NIST) XCOM, respectively [17], [22], [23].

Several studies have explored the possibility of BMD estimation on diagnostic CT, i.e. not CT for RT, but the lack of standardized calibration phantoms and CT settings has made transferability between centres challenging [10], [11], [13], [15], [24]. Accurate BMD estimation requires stable CT numbers, and RT CT scans meet these standards, which make them suitable for this purpose. To the best of our knowledge, only one other study has used RT CT for BMD estimation [14]. However, that study relied on a method where CT numbers were converted to quantitative BMD values using a predefined calibration formula. The theoretically derived method presented here is independent of CT device and phantom calibration, enabling use in clinical practice, multicentre studies, and retrospective analyses. Awareness of CT number uncertainties is crucial, since BMD estimates are translated directly from these numbers. Therefore, for future implementation of this method, it is strongly recommended to optimize CT scanner settings to ensure CT number stability. Verification of the method using trabecular bone inserts at the local site is also advised prior to clinical implementation [24].

The study results were validated using a spine phantom, and the estimated BMD aligned well with manufacturer specifications. Due to the spine phantom design, there were no overlying structures, and the homogeneous surrounding material provided optimal conditions for reliable DXA results. Comparing CT-based BMD with DXA confirmed that the developed method performed well.

A key consideration was the method’s applicability using different CT devices, as scanners may differ in both hardware and software. A high agreement was found in BMD estimation between the CT scanners used here, SOMATOM and Aquilion, and the difference was considered small (5.9 mg/cm^3^) and clinically irrelevant. One distinction between the two CT scanners was the beam hardening correction of SOMATOM providing more stable CT numbers less affected by scan volume [25]. The CT numbers were indeed consistent for small and medium phantoms. For the largest phantom (W 52 cm, H 36.6 cm), corresponding to a body circumference of severe obesity, slightly larger variations were seen. The influence of body size on BMD estimation by CT needs further investigation.

The method was evaluated for retrieving CT numbers from patient data, and it was found that one VOI was easier to delineate than three ROIs, while also providing more data points, potentially making it more robust. Similar trabecular bone delineation methods have been tested in other studies of opportunistic osteoporosis screening using diagnostic CT [10], [11], [12], [13], [14], [15]. Further, a larger VOI covering the entire vertebral body (trabecular and cortical bone) was tested to simulate DXA conditions, but this approach proved to be time-consuming and subjective. Consequently, the mean of four spherical VOIs centrally placed in Th9–Th12 was used to account for vertebral variation, which showed an excellent interobserver variability.

The internal calibration method is a more comprehensive approach since multiple structures need to be delineated, and the choice of internal materials for calibration must be made. Air/fat and air/blood combinations were tested, and differences in the results were observed depending on which material was used. For example, blood may contain calcifications, especially in older women.

Normal reference intervals for vertebral BMD (mg/cm^3^) estimated from CT images are still lacking [12], despite several studies on CT-based BMD estimation [10], [11], [12], [13], [14], [15]. Osteoporosis thresholds have been suggested but are not established [15]. Future work will address BMD variation within the spine and will define trabecular BMD cut-off levels. The DXA method is still the standard for diagnosing osteoporosis, and a systematic CT–DXA comparison is needed to assess the clinical value of CT-based BMD.

The derived method is currently limited to the lower thoracic vertebrae. Specific CT scanner dependencies that influence CT number accuracy require further evaluation. Additionally, the CT numbers depend on how the VOI is defined, including which vertebrae and subregions are excluded. As always, it is necessary to validate how well the method performs in combination with the local equipment before clinical implementation.

In conclusion, CT images acquired for RT planning can be used for BMD assessment without requiring additional imaging or radiation exposure. The theoretically derived HU-to-BMD translation function has been validated with phantoms and internal calibration, demonstrating reliable performance. The trabecular bone delineation technique was simple and reproducible. The long-term goal is to implement this method as a tool for BMD assessment in cancer patients receiving RT, and future work will focus on further validation and defining normal BMD cut-off levels.

## CRediT authorship contribution statement

**Julia Götstedt:** Writing – review & editing, Writing – original draft, Visualization, Validation, Supervision, Software, Resources, Project administration, Methodology, Investigation, Formal analysis, Data curation, Conceptualization. **Frida Ljung:** Writing – review & editing, Visualization, Software, Investigation, Formal analysis, Data curation. **Jenny Nilsson:** Writing – review & editing, Validation, Investigation. **Erik Pettersson:** Writing – review & editing, Validation, Supervision, Software, Methodology, Formal analysis, Data curation. **Sofia Heyman:** Writing – review & editing, Writing – original draft, Visualization, Validation, Supervision, Software, Resources, Project administration, Methodology, Investigation, Funding acquisition, Formal analysis, Data curation, Conceptualization.

## Funding

Varian Medical Systems; the King Gustaf V Jubilee Clinic Cancer Research Foundation, Grant (2023/503; 2026); The Gothenburg Society of Medicine, GLS-1000085.

## Declaration of competing interest

The authors declare that they have no known competing financial interests or personal relationships that could have appeared to influence the work reported in this paper.
