# Supplementary material for: Estimation of bone mineral density with computed tomography performed in radiotherapy planning
Source: Phys Imaging Radiat Oncol. 2026 May 3;39:100986. doi: 10.1016/j.phro.2026.100986 (PMC13186006; doi:10.1016/j.phro.2026.100986)
Supplement: Supplementary Data 1 [file mmc1.docx]

## Supplementary material A

### Theoretical determination of radiodensity and bone mineral density

Data were retrieved for elemental composition, mass density (*ρ_t_*), and elemental mass fraction (*w_i_*) for the reference bone specimens (Table S1) [1]. The mass attenuation coefficients (*µ*/*ρ*) at 71 keV for each element (n = 11) composing bone tissue and water (*µ_w_*) were retrieved (Table S2) [2]. This photon energy (71 keV) was chosen, since it corresponded most closely to the 120 kV X-ray spectrum used on the CT scanners in this study.

The attenuation coefficient of the reference bone tissue (*µ_t_*) was calculated by using the Bragg’s additivity rule (Eq. 1) [3]:

$$\begin{aligned} \mu_{t}=\rho_{t}\sum_{i=1}^{N} w_{i}\left( \frac{\mu}{\rho} \right)_{i}\#\left( 1 \right) \end{aligned}$$

Radiodensity (Hounsfield units; HU) was calculated using Eq. 2 [4]:

$$\begin{aligned} CT number=1000\times\left( \frac{\mu_{t}}{\mu_{w}}-1 \right)\#\left( 2 \right) \end{aligned}$$

Calcium content (*w_Ca_*) in pure bone mineral (calcium hydroxyapatite; CaHA) is constant, *w*_Ca_ (CaHA) = 0.399 [5]. Bone mineral density (BMD; mg/cm^3^) was calculated by assuming that all calcium present in the bone tissue was bound to bone mineral (Eq. 3):

$$\begin{aligned} BMD=\frac{\left( \rho_{t}\times w_{Ca} \right)}{0.399}\#\left( 3 \right) \end{aligned}$$

## Supplementary material B

### CT scanners and CT protocols

The CT scans by SOMATOM go.Open Pro (Siemens Healthineers, Germany) were performed according to standard protocols: tube voltage 120 kV, kernel Qr40f, scan field of view (SFOV) 600 mm, bow-tie filter type W1, scan slice thickness of 2 mm. The kernel used (Qr40f) and iterative bone beam hardening correction (iBHC) ensured stable CT numbers independent of body constitution [6].

The CT scans by Aquilion LB (Toshiba Medical Systems Corporation, Japan) were performed according to standard protocols: tube voltage 120 kV, kernel FC17, scan slice thickness of 2 mm, SFOV 550-700 mm. The FC17 kernel is used for high-resolution reconstructions and is typically applied to depict bone and is suitable for assessing bone mineral density [7]. The Aquilion scanner used did not have the iBHC feature.

Table S1. Bone mineral density (BMD) of reference specimens comprising trabecular bone.

| **Tissue** | **Mass density** (mg/cm^3^) | **Ca mass fraction** | **Ca density**  **(**mg/cm^3^) | **BMD**  (mg/cm^3^) |
| --- | --- | --- | --- | --- |
| **Bone Mineral** | 3225 | 0.399 | 1 286.8 | 3 225.0 |
| **Bone specimens from female** | | | | |
| Humerus (proximal) | 1185 | 0.069 | 81.8 | 204.9 |
| Humerus (distal)^1^ | 1117 | 0.052 | 58.1 | 145.6 |
| Clavicles | 1192 | 0.071 | 84.6 | 212.1 |
| Cranium | 1252 | 0.088 | 110.2 | 276.1 |
| Femur (proximal) | 1046 | 0.020 | 20.9 | 52.4 |
| Mandible | 1189 | 0.069 | 82.0 | 205.6 |
| Pelvis | 1105 | 0.039 | 43.1 | 108.0 |
| Ribs | 1087 | 0.028 | 30.4 | 76.3 |
| Scapulae | 1125 | 0.048 | 54.0 | 135.3 |
| Cevical vertebrae | 1129 | 0.044 | 49.7 | 124.5 |
| Thoracic vertebrae | 1080 | 0.025 | 27.0 | 67.7 |
| Lumbar vertebrae | 1165 | 0.057 | 66.4 | 166.4 |
| Sacrum | 1052 | 0.014 | 14.7 | 36.9 |
| Sternum | 1073 | 0.022 | 23.6 | 59.2 |
| **Bone specimens from male** | | | | |
| Humerus (proximal) | 1233 | 0.083 | 102.3 | 256.5 |
| Humerus (distal)^1^ | 1109 | 0.049 | 54.3 | 136.2 |
| Clavicles | 1157 | 0.060 | 69.4 | 174.0 |
| Cranium | 1165 | 0.062 | 72.2 | 181.0 |
| Femur (proximal) | 1125 | 0.050 | 56.3 | 141.0 |
| Mandible | 1271 | 0.093 | 118.2 | 296.2 |
| Pelvis | 1121 | 0.045 | 50.4 | 126.4 |
| Ribs | 1170 | 0.058 | 67.9 | 170.1 |
| Scapulae | 1201 | 0.073 | 87.7 | 219.7 |
| Cevical vertebrae | 1049 | 0.012 | 12.6 | 31.5 |
| Thoracic vertebrae | 1070 | 0.021 | 22.5 | 56.3 |
| Lumbar vertebrae | 1108 | 0.036 | 39.9 | 100.0 |
| Sacrum | 1033 | 0.006 | 6.2 | 15.5 |
| Sternum | 1041 | 0.001 | 1.0 | 2.6 |

^1^ also including ulnae and radii, wrists and hand bones, femora, lower, tibiae, fibulae and patellae, ankles and foot, spongiosa. Ca, calcium; BMD, bone mineral density.

Table S2. Simulated CT values for fat and bone tissue. The bone specimen comprised trabecular bone. Attenuation at mono-energetic photon energy of 71 keV was simulated based on information of mass density and elemental composition of the reference tissues.

|  | **Mass density**  **(mg/cm^3^)** | **Elemental proportion by weight** | | | | | | | | | | | | | | | | | | | | | | | | | **CT value (HU)** |
| --- | --- | --- | --- | --- | --- | --- | --- | --- | --- | --- | --- | --- | --- | --- | --- | --- | --- | --- | --- | --- | --- | --- | --- | --- | --- | --- | --- |
|  |  | **H** | | **C** | | **N** | | **O** | | **Na** | | **Mg** | | **P** | | **S** | | **Cl** | | **K** | | **Ca** | | **Fe** | | **Zn** |  |
| **Fat^1^** | 920 | 0.119 | 0.637 | | 0.008 | | 0.232 | | 0.001 | | 0.000 | | 0.000 | | 0.001 | | 0.001 | | 0.000 | | 0.000 | | 0.000 | | 0.00002 | | -101.300 |
| **Bone specimen from female** | | | | | | | | | | | | | | | | | | | | | | | | | | | |
| Humerus (proximal) | 1185 | 0.086 | 0.392 | | 0.026 | | 0.390 | | 0.001 | | 0.001 | | 0.031 | | 0.002 | | 0.001 | | 0.001 | | 0.069 | | 0 | | 0 | | 283.965 |
| Humerus (distal)^1^ | 1117 | 0.095 | 0.498 | | 0.017 | | 0.311 | | 0.001 | | 0.000 | | 0.023 | | 0.002 | | 0.001 | | 0 | | 0.052 | | 0 | | 0 | | 177.792 |
| Clavicles | 1192 | 0.085 | 0.388 | | 0.026 | | 0.392 | | 0.001 | | 0.001 | | 0.032 | | 0.002 | | 0.001 | | 0.001 | | 0.071 | | 0 | | 0 | | 295.046 |
| Cranium | 1252 | 0.079 | 0.345 | | 0.029 | | 0.413 | | 0.002 | | 0.001 | | 0.039 | | 0.002 | | 0.001 | | 0.001 | | 0.088 | | 0 | | 0 | | 393.397 |
| Femur (proximal) | 1046 | 0.104 | 0.501 | | 0.019 | | 0.342 | | 0.001 | | 0.000 | | 0.009 | | 0.002 | | 0.001 | | 0.001 | | 0.020 | | 0 | | 0 | | 53.920 |
| Mandible | 1189 | 0.086 | 0.383 | | 0.027 | | 0.398 | | 0.001 | | 0.001 | | 0.031 | | 0.002 | | 0.001 | | 0.001 | | 0.069 | | 0 | | 0 | | 288.804 |
| Pelvis | 1105 | 0.096 | 0.422 | | 0.025 | | 0.394 | | 0.001 | | 0.000 | | 0.018 | | 0.002 | | 0.001 | | 0.001 | | 0.039 | | 0.001 | | 0 | | 150.751 |
| Ribs | 1087 | 0.098 | 0.394 | | 0.029 | | 0.431 | | 0.001 | | 0.000 | | 0.013 | | 0.002 | | 0.002 | | 0.001 | | 0.028 | | 0.001 | | 0 | | 115.417 |
| Scapulae | 1125 | 0.093 | 0.426 | | 0.024 | | 0.382 | | 0.001 | | 0.000 | | 0.022 | | 0.002 | | 0.001 | | 0.001 | | 0.048 | | 0 | | 0 | | 182.545 |
| Cevical vertebrae | 1129 | 0.092 | 0.371 | | 0.030 | | 0.436 | | 0.001 | | 0.000 | | 0.020 | | 0.002 | | 0.002 | | 0.001 | | 0.044 | | 0.001 | | 0 | | 185.556 |
| Thoracic vertebrae | 1080 | 0.098 | 0.399 | | 0.029 | | 0.430 | | 0.001 | | 0.000 | | 0.012 | | 0.002 | | 0.002 | | 0.001 | | 0.025 | | 0.001 | | 0 | | 102.619 |
| Lumbar vertebrae | 1165 | 0.088 | 0.352 | | 0.031 | | 0.440 | | 0.001 | | 0.001 | | 0.026 | | 0.002 | | 0.001 | | 0.001 | | 0.057 | | 0 | | 0 | | 242.553 |
| Sacrum | 1052 | 0.102 | 0.416 | | 0.028 | | 0.426 | | 0.001 | | 0.000 | | 0.007 | | 0.002 | | 0.002 | | 0.001 | | 0.014 | | 0.001 | | 0 | | 56.462 |
| Sternum | 1073 | 0.100 | 0.403 | | 0.028 | | 0.429 | | 0.001 | | 0.000 | | 0.011 | | 0.002 | | 0.002 | | 0.001 | | 0.022 | | 0.001 | | 0 | | 91.548 |
| **Bone specimen from male** | | | | | | | | | | | | | | | | | | | | | | | | | | | |
| Humerus (proximal) | 1233 | 0.081 | 0.354 | | 0.028 | | 0.410 | | 0.002 | | 0.001 | | 0.037 | | 0.002 | | 0.001 | | 0.001 | | 0.083 | | 0 | | 0 | | 363.161 |
| Humerus (distal)^1^ | 1109 | 0.096 | 0.504 | | 0.017 | | 0.308 | | 0.001 | | 0.000 | | 0.022 | | 0.002 | | 0.001 | | 0 | | 0.049 | | 0 | | 0 | | 164.345 |
| Clavicles | 1157 | 0.089 | 0.409 | | 0.025 | | 0.385 | | 0.001 | | 0.000 | | 0.027 | | 0.002 | | 0.001 | | 0.001 | | 0.060 | | 0 | | 0 | | 237.223 |
| Cranium | 1165 | 0.088 | 0.395 | | 0.026 | | 0.395 | | 0.001 | | 0.001 | | 0.028 | | 0.002 | | 0.001 | | 0.001 | | 0.062 | | 0 | | 0 | | 249.991 |
| Femur (proximal) | 1125 | 0.093 | 0.441 | | 0.023 | | 0.365 | | 0.001 | | 0.001 | | 0.022 | | 0.002 | | 0.001 | | 0.001 | | 0.050 | | 0 | | 0 | | 185.299 |
| Mandible | 1271 | 0.077 | 0.332 | | 0.030 | | 0.420 | | 0.002 | | 0.001 | | 0.041 | | 0.002 | | 0.001 | | 0.001 | | 0.093 | | 0 | | 0 | | 424.170 |
| Pelvis | 1121 | 0.094 | 0.409 | | 0.026 | | 0.400 | | 0.001 | | 0.001 | | 0.020 | | 0.002 | | 0.001 | | 0.001 | | 0.045 | | 0 | | 0 | | 174.190 |
| Ribs | 1170 | 0.088 | 0.346 | | 0.031 | | 0.444 | | 0.001 | | 0.001 | | 0.026 | | 0.002 | | 0.001 | | 0.001 | | 0.058 | | 0.001 | | 0 | | 253.745 |
| Scapulae | 1201 | 0.084 | 0.373 | | 0.027 | | 0.404 | | 0.001 | | 0.001 | | 0.033 | | 0.002 | | 0.001 | | 0.001 | | 0.073 | | 0 | | 0 | | 308.979 |
| Cevical vertebrae | 1049 | 0.103 | 0.416 | | 0.028 | | 0.428 | | 0.001 | | 0.000 | | 0.006 | | 0.002 | | 0.002 | | 0.001 | | 0.012 | | 0.001 | | 0 | | 50.584 |
| Thoracic vertebrae | 1070 | 0.100 | 0.403 | | 0.028 | | 0.431 | | 0.001 | | 0.000 | | 0.010 | | 0.002 | | 0.002 | | 0.001 | | 0.021 | | 0.001 | | 0 | | 86.379 |
| Lumbar vertebrae | 1108 | 0.095 | 0.380 | | 0.030 | | 0.436 | | 0.001 | | 0.000 | | 0.016 | | 0.002 | | 0.002 | | 0.001 | | 0.036 | | 0.001 | | 0 | | 150.085 |
| Sacrum | 1033 | 0.105 | 0.426 | | 0.027 | | 0.426 | | 0.001 | | 0.000 | | 0.003 | | 0.002 | | 0.002 | | 0.001 | | 0.006 | | 0.001 | | 0 | | 24.849 |
| Sternum | 1041 | 0.104 | 0.421 | | 0.028 | | 0.427 | | 0.005 | | 0.000 | | 0.002 | | 0.002 | | 0.001 | | 0.009 | | 0.001 | | 0 | | 0 | | 30.187 |

^1^also including ulnae and radii, wrists and hand bones, femora, lower, tibiae, fibulae and patellae, ankles and foot. H, hydrogen; C, carbon; N, nitrogen; O, oxygen; Na, sodium; Mg, magnesium; P, phosphorus; S, sulfur; Cl, clorid; K, potassium; Ca, calcium; Fe, iron; Zn, zink; HU, Hounsefield unit.

## References

[1] Kim CH, Yeom YS, Petoussi-Henss N, Zankl M, Bolch WE, Lee C, et al. Adult mesh-type reference computational phantoms. Annals of the ICRP. 2020;49:ANNEX B: Table B.1 (male) och B.2 (female), specimen no 7-20. [https://doi.org/10.1177/0146645319893605](https://journals.sagepub.com/doi/10.1177/0146645319893605).

[2] National Institute of Standards and Technology. Tables of X-Ray Mass Attenuation Coefficients and Mass Energy Absorption Coefficients (version 1.4). [http://physics.nist.gov/xaamdi](https://www.nist.gov/pml/x-ray-mass-attenuation-coefficients); 2024 [accesssed 19 Dec 2025].

[3] Thwaites DI. Bragg's Rule of Stopping Power Additivity: A Compilation and Summary of Results. Radiation Research. 1983;95:495-518. <https://doi.org/10.2307/3576096>.

[4] Levine ZH, Peskin AP, Holmgren AD, Garboczi EJ. Preliminary X-ray CT investigation to link Hounsfield unit measurements with the International System of Units (SI). PLOS ONE. 2018;13:e0208820. <https://doi.org/10.1371/journal.pone.0208820>.

[5] White DR, Griffith RV, Wilson IJ. ICRU Report-46: Photon, electron, proton and neutron interaction data for body tissues. J ICRU. 1992;os-24:5-13. <https://doi.org/10.1093/jicru_os24.1.1>.

[6] Pettersson E, Norrlid O, Dahlgren C, Bäck A. Does patient size affect the CT-numbers of bones? Radiother Oncol. 2024;194:3815-7. <https://doi.org/10.1016/S0167-8140(24)01430-0>.

[7] Ivanov D, Kirillova I, Kossovich L, Bessonov L, Petraikin A, Dol AV, et al. Influence of convolution kernel and beam-hardening effect on the assessment of trabecular bone mineral density using quantitative computed tomography. Izvestiya of Saratov University New Series Series: Mathematics Mechanics Informatics. 2020;20:205-19. <https://doi.org/10.18500/1816-9791-2020-20-2-205-219>.
